# Microextraction of Tigecycline Using Deep Eutectic Solvents and Its Determination in Milk by LC-MS/MS Method

**DOI:** 10.1021/acs.jafc.3c03023

**Published:** 2023-07-24

**Authors:** Ilona Kiszkiel-Taudul, Patrycja Stankiewicz

**Affiliations:** Chemical Department, University of Bialystok, 15-245 Bialystok, Poland

**Keywords:** tigecycline, antibacterial agents, hydrophobic deep eutectic solvents, liquid−liquid microextraction, liquid chromatography, tandem mass spectrometry, dairy products

## Abstract

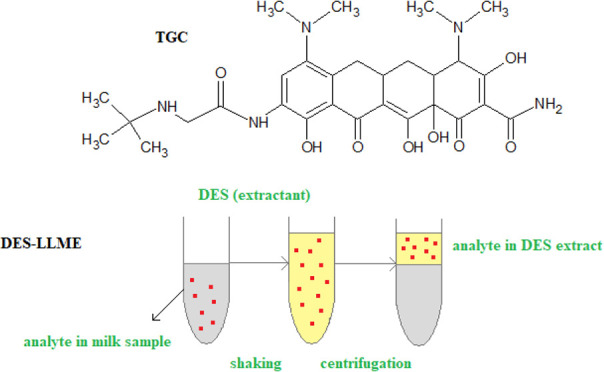

The occurrence of tigecycline (TGC), a new first glycylcycline antibiotic residues in food products harmfully influences potential human consumers health. Therefore, analysts are forced to develop new microextraction methods connected with modern extractants for effective isolation of this compound. For this purpose, deep eutectic solvents (DES) as the extraction media were used. Liquid–liquid microextraction (LLME) of tigecycline from milk samples with application of the hydrophobic deep eutectic solvents: decanoic acid:thymol (1:1), thymol:camphor (2:1), dodecanoic acid:menthol (2:1), and dodecanoic acid:dodecanol (1:1) was developed. The studied samples were subjected to a deproteinization process using trichloroacetic acid solution and acetonitrile. The optimal microextraction parameters, molar ratio of DES components, amount of extraction solvents, pH of milk sample, shaking, and centrifugation time, were chosen. Tigecycline in the obtained microextracts of deep eutectic solvents was analyzed using a liquid chromatographic technique connected with a tandem mass spectrometry (LC-MS/MS) system. The limits of detection and quantification values for TGC determination followed by DES-LLME-LC-MS/MS method were in the 1.8 × 10^–11^ mol L^–1^ (0.01 μg kg^–1^) to 4.0 × 10^–9^ mol L^–1^ (2.28 μg kg^–1^) and 5.5 × 10^–11^ mol L^–1^ (0.03 μg kg^–1^) to 1.2 × 10^–8^ mol L^–1^ (6.84 μg kg^–1^) ranges, respectively. The RSD values of precision were in the range 1.4–7.8% (intraday) and 5.4–11.7% (interday). The developed procedures were used for the determination of tigecycline in different bovine milk samples.

## 1 Introduction

**Figure 1:**
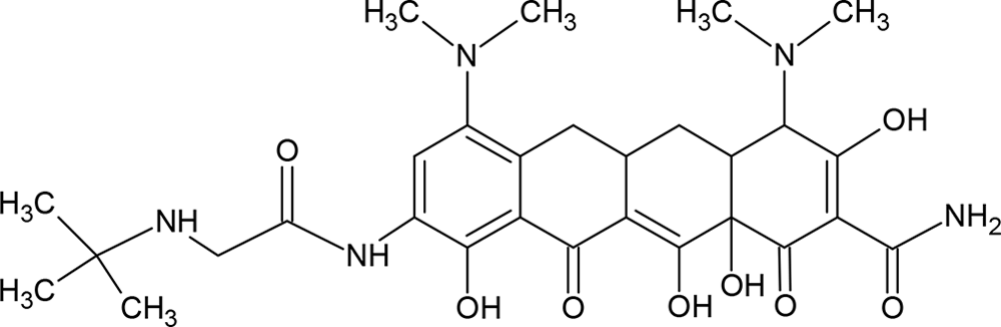
Molecular structure of tigecycline (TGC).

Tigecycline (TGC) is the first antibiotic from the glycylcycline group (tetracycline class). This antibacterial drug contains *tert*-butyl-glycylamido side chain in the aromatic ring (Figure 1).^1,2^ The discussed compound as a new generation bacteriostatic antibiotic is used in infections caused by Gram-positive, Gram-negative, and anaerobic bacteria to treat skin, soft tissue, abdominal cavity, and acquired pneumonia. Tigecycline was approved by the U.S. Food and Drug Administration (FDA) in 2005. The necessity for the use of new antibacterial agents in different diseases is due to the emergence of multidrugresistant bacteria. Tigecycline is administrated at a standard dose of 100 mg (50 mg at 12 h intervals). The main metabolites of this antibiotic are tigecycline glucuronide, epimer of tigecycline glucuronide and *N*-acetyl-9-aminominocycline.^3−6^

The use of antibiotics in veterinary medicine causes their occurrence in foods of animal origin. The residues of antibacterial drugs in milk harmfully influence consumer health (e.g., allergic reactions, gastrointestinal disturbance). Therefore, the presence and amount of antibiotics in dairy products should be monitored. The analysts are forced to develop new methods enabling the effective isolation and selective determination of these compounds. The elaborated procedures should be especially enable detection of the new generation antibiotics (including tigecycline).^7,8^

Currently, the deep eutectic solvents (DES) are used as extraction media for isolation of analytes from the different matrices including milk samples after the deproteinization process.^9−14^ These extractants are composed of HBA hydrogen bond acceptors (e.g., quaternary ammonium salts) and HBD hydrogen bond donors (e.g., carboxylic acids, alcohols, and amines). The melting point of DES is lower than the same parameter for each individual component (HBA and HBD). Hydrophobic deep eutectic solvents have been acknowledged as a new class of green solvents which may replace traditional organic solvents in liquid–liquid extraction and also ionic liquids.^15−19^ These extraction media exhibit unique properties, such as nonflammability, negligible vapor pressure, thermal stabilities, and low volatilities. Additionally, DES has the advantages in comparison to ionic liquids, namely, low cost, easy preparation, and production from nontoxic and biocompatible materials.^20,21^

The use of deep eutectic solvents in the liquid–liquid microextraction process (LLME) gives the possibility of the amount reducing of the extractants. The partition of analytes from sample to DES microdrops enables enrichment of the studied compounds.^22−24^ The microextraction processes using deep eutectic solvents as extractants may be environmentally friendly alternatives to the classical procedures with organic solvents for antibiotics and the other analytes isolation.^25−29^ The connection of miniaturized isolation procedures using DES as extraction media and liquid chromatography coupled with tandem mass spectrometry (LC-MS/MS) method ensures sensitive determination of analytes at low level concentration (ng L^–1^ or μg L^–1^).^30,31^ According to our knowledge, the microextraction procedure using deep eutectic solvents has not been applied for the isolation of tigecycline from milk samples.

**Figure 2:**
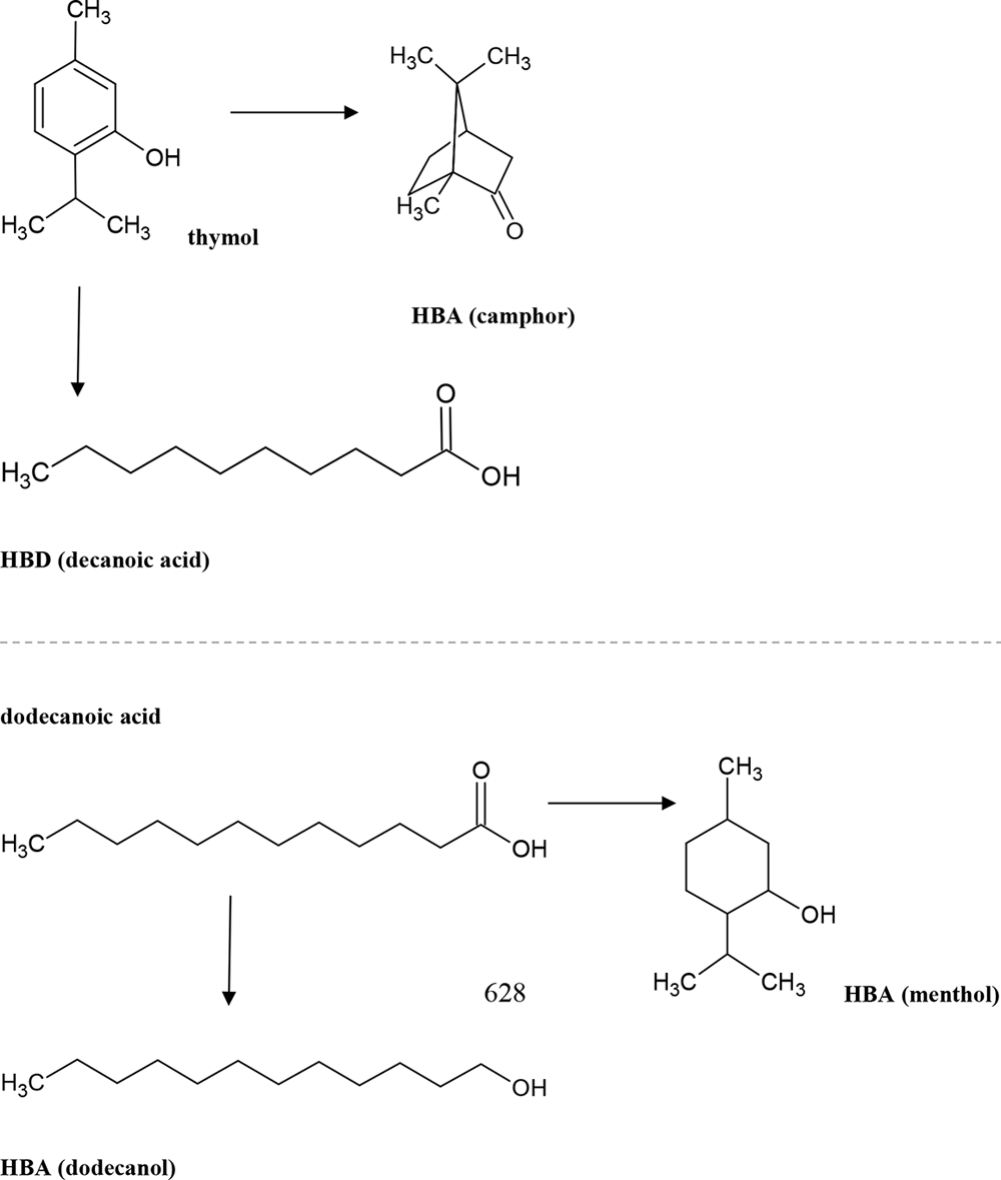
Molecular structures of deep eutectic solvent components (hydrogen bond donors and acceptors).

The presented paper describes developed “green” microextraction procedures for the isolation of tygecycline from milk samples. The isolation process of analyte was performed using deep eutectic solvents thymol:camphor, thymol:decanoic acid, dodecanoic acid:menthol, and dodecanoic acid:dodecanol as extractants (DES-LLME) (Figure 2). The obtained extracts were analyzed using a liquid chromatography method connected with tandem mass spectrometry (LC-MS/MS).

## 2 Experimental Section

### 2.1 Apparatus

A vortex mixer (Heidolph Vibramax 110, Germany) and a centrifuge (MPW-251, Poland) were used for liquid–liquid microextraction. The chromatographic measurements were done by applying a liquid chromatography–tandem mass spectrometry system (Shimadzu LC-MS/MS-8040, Japan) consisted of a triple quadrupole mass spectrometer with turbo ion spray ionization source in the positive ion mode, pump (Shimadzu LC-30AD, Japan), thermostat column oven (Shimadzu CTO-20AC, Japan), autosampler (Shimadzu SIL-30AC, Japan), degasser (Shimadzu DGU-20A5R, Japan), and nitrogen generator (Shimadzu Peak Scientific NM32LA, Japan).

### 2.2 Reagents and Solutions

The standard solution of tigecycline (1 × 10^–3^ mol L^–1^) was prepared by dissolving an appropriate weighed amount of the active substance (USP, China) in 100 mL of doubly distilled water. The deep eutectic solvents were prepared by mixing thymol (Sigma-Aldrich, India) and camphor (Sigma-Aldrich, China), thymol and decanoic acid (Sigma-Aldrich, Malaysia), dodecanoic acid (Sigma-Aldrich, Malaysia) and menthol (Sigma-Aldrich, Germany), and dodecanoic acid and dodecanol (Sigma-Aldrich, USA) in a suitable mass ratio and stirring this mixtures at 40 °C until formation of the clear liquids.

Acetonitrile (purity HPLC), water, methanol, and 98% formic acid (purity LC-MS) were obtained from Honeywell (USA) and Merck (Germany). Trichloroacetic acid, 35–38% (w/w) hydrochloric acid, and sodium hydroxide were supplied from POCH SA (Poland). Stock solutions of trichloroacetic acid (0.7 mol L^–1^), hydrochloric acid (0.1 mol L^–1^), formic acid (0.1% v/v), and sodium hydroxide (0.1 mol L^–1^) were prepared by dissolving appropriate amounts in 500 mL of doubly distilled water.

### 2.3 Milk Samples Preparation

The deproteinization process of 2.0% (w/w) milk samples (purchased in local markets, Bialystok, Poland) was performed as follows: 5 mL of milk sample spiked with 300 μL of tigecycline solution (1 × 10^–3^ mol L^–1^) was transferred into a 15 mL centrifuge tube. Then 5 mL of trichloroacetic acid (0.7 mol L^–1^) or acetonitrile was added to the milk sample before DES-LLME isolation. The solution of trichloroacetic acid was used for the deproteinization process before microextraction with thymol:decanoic acid and thymol:camphor as extractants, whereas acetonitrile solvent was applied during the deproteinization of the spiked milk sample before the isolation process of tigecycline using DES dodecanoic acid:menthol, dodecanoic acid:dodecanol. The mixtures were shaken on a vortex mixer for 5 min at 1500 rpm and centrifuged for 10 min at 5000 rpm. The supernatants were filtered through a paper filter. The final concentration of tygecycline was calculated to be 3 × 10^–5^ mol L^–1^.

### 2.4 Extraction Procedures of Tigecycline (DES-LMME)

#### 2.4.1 Liquid–Liquid Microextraction Using Deep Eutectic Solvents with Thymol

For the microextraction process of TGC from spiked milk sample, 5 mL of supernatant containing analyte after the deproteinization process (procedure 2.3 using trichloroacetic acid solution) was transferred into a 15 mL centrifuge tube. Then 300 μL of deep eutectic solvent consisting of thymol and decanoic acid at a mass ratio of 1:1, which was prepared according to the procedure 2.2, was added. The content was shaken on the vortex mixer for 30 min at 1500 rpm and centrifuged (10 min, 2600 rpm). After the separation of phases, the obtained extracts were analyzed by the chromatographic analysis LC-MS/MS.

Microextraction of tigecycline from milk samples by DES consisting of camphor and thymol at a mass ratio of 1:2 was performed using 200 μL of deep eutectic solvent. Then the extractive sample was shaken for 20 min at 1250 rpm and centrifuged (5 min, 5000 rpm) before chromatographic analysis.

#### 2.4.2 Liquid–Liquid Microextraction Using Deep Eutectic Solvents with Dodecanoic Acid

The isolation process of tigecycline was performed for a spiked milk sample using DES with dodecanoic acid. After the deproteinization process (procedure 2.3 using acetonitrile), the supernatant was transferred into a 15 mL centrifuge tube. Then 700 μL of deep eutectic solvent prepared according to procedure 2.2 and consisting of menthol and dodecanoic acid at a mass ratio of 1:2 was added. The sample was shaken on the vortex mixer for 20 min at 1500 rpm and centrifuged for 10 min at 4000 rpm. The separation of phases was achieved, and the analysis of the obtained extracts was performed using LC-MS/MS method.

Microextraction of TGC from the milk sample by deep eutectic solvent consisting of dodecanoic acid and dodecanol at a mass ratio of 1:1 was performed using 700 μL of extractant. The shaking time of the extractive sample was equal to 20 min (1000 rpm) and centrifugation time 5 min (5000 rpm). After that, chromatographic analysis of the extracts was performed.

### 2.5 Chromatographic Analysis of Tigecycline with LC-MS/MS Technique

The LC-MS/MS analysis of tigecycline after microextraction using deep eutectic solvents with thymol was performed on a Kinetex C-18 (50 mm × 2.1 mm, 1.7 μm) column using a mobile phase consisting of 0.1% formic acid and methanol (1:1 v/v) at a flow rate of 0.4 mL min^–1^. The injection volume was 5 μL. The total run time was equal 5 min and characteristic peak of TGC was observed at retention time of 0.405 and 0.437 min for extracts of DES thymol:decanoic acid and camphor:thymol, respectively.

The chromatographic analysis of TGC after isolation process using deep eutectic solvents with dodecanoic acid was performed on a Kinetex PFP (50 mm × 2.1 mm, 1.7 μm) column using a mobile phase consisting of 0.1% formic acid and (methanol/acetonitrile 2:3 v/v) (1:1 v/v) at a flow rate of 0.4 mL min^–1^. The injection volume was 5 μL. The total run time was equal 5 min, and a characteristic peak of tigecycline was observed at retention time of 0.336 and 0.327 min for extracts of DES menthol:dodecanoic acid and dodecanoic acid:dodecanol, respectively.

The parameters of mass spectrometer analysis were as follows: the collision gas (argon), collision cell gas pressure 230 Pa, the flow rate of drying gas (nitrogen) 15 L min^–1^, and nebulizing gas (nitrogen) 3 L min^–1^. Multiple reaction monitoring (MRM) mode was used to study parent → product ions (*m*/*z*) transitions for tigecycline in ESI positive ionization: 586.30 → 569.25 (collision energy 21 V), 586.30 → 513.20 (collision energy 29 V), and 586.30 → 456.15 (collision energy 36 V).

## 3 Results and Discussion

### 3.1 Primary Studies (Selection of DES Type)

The deep eutectic solvents, so-called “green solvents” and their use during liquid–liquid microextraction create environmentally friendly isolation methods. Therefore, the different hydrophobic deep eutectic solvents were prepared by mixing of the hydrogen bond acceptors and hydrogen bond donors in the mass ratio 1:1 and were stirred at 40 °C. The components of DES that have formed clear liquids are presented in Table 1 and were used for microextraction process of tigecycline. It was observed that effective isolation of the studied analyte using deep eutectic solvents consisted of thymol as HBD and camphor (HBA) or thymol as HBA and decanoic acid (HBD). The proper phase separation during the microextraction process was achieved with the use of the mentioned DES as extraction media for milk samples after the deproteinization process using trichloroacetic acid solution, whereas the application of DES consisting of dodecanoic acid as HBD and menthol (HBA) or dodecanoic acid as HBD and dodecanol (HBA) enabled effective microextraction of TGC from milk samples after the deproteinization process using acetonitrile solvent.

**Table 1:** Composition of HBA and HBD Components to Create Hydrophobic Deep Eutectic Solvents Used in Microextraction of Tigecycline from Milk Samples

hydrogen bond acceptor (HBA)	hydrogen bond donor (HBD)
tetrabutylammonium bromide	dodecanoic acid, decanoic acid, octanoic acid, undecanol, decanol, dodecanol, thymol, menthol
methyltrioctylammonium chloride	thymol, octanoic acid, undecanol, decanol, dodecanol, menthol
camphor	thymol, octanoic acid, decanoic acid

thymol	octanoic acid, decanoic acid, dodecanoic acid
menthol	octanoic acid, decanoic acid, dodecanoic acid

decanol	dodecanoic acid
dodecanol	dodecanoic acid

### 3.2 Choosing Conditions of LC-MS/MS Analysis

**Figure 3:**
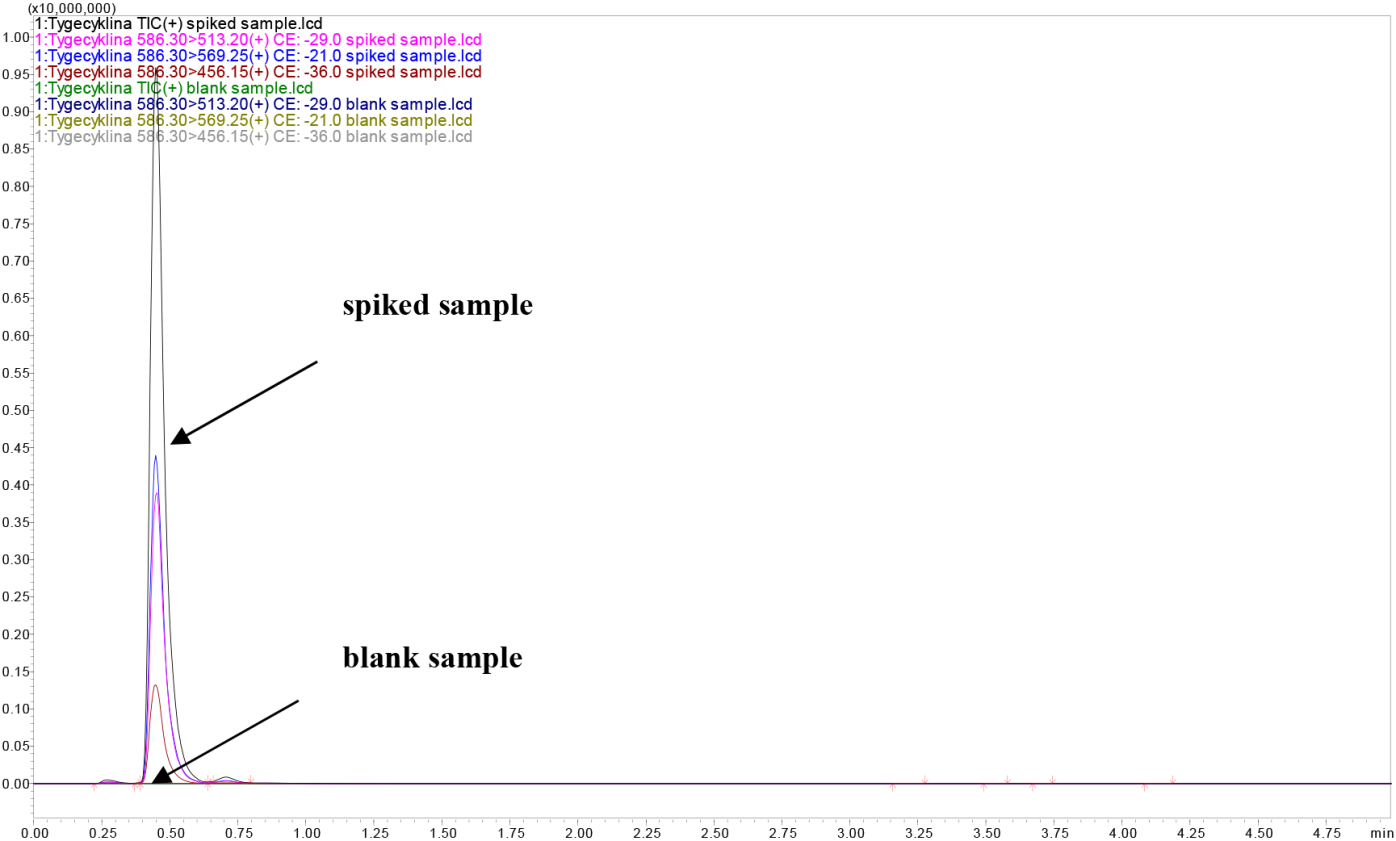
Chromatogram MRM of a blank milk sample and spiked milk sample with analyte after microextraction using DES thymol:camphor.

The obtained deep eutectic solvent extracts containing tigecycline were analyzed using a chromatographic method connected with tandem mass spectrometry. The different columns: phenyl–hexyl, PFP, C-18, and mobile phases consisting of 0.1% formic acid and methanol or acetonitrile in the different ratios: 1:4 v/v, 1:2 v/v, 1:1 v/v, 2:1 v/v, and 4:1 v/v were studied during determination of TGC. The mass spectrometer worked in ESI positive ionization under the multiple reaction monitoring (MRM). It was found that the DES extracts after the deproteinization process using trichloroacetic acid solution should be analyzed on C-18 column and mobile phase 0.1% formic acid/methanol (1:1 v/v), whereas the obtained extracts with tigecycline after the deproteinization by acetonitrile solvent were studied using PFP column and a mobile phase consisting of 0.1% formic acid and (methanol/acetonitrile 2:3 v/v) (1:1 v/v). The characteristic peak of the studied compound was observed in the range 0.327–0.437 min depending on the kind of the deep eutectic solvents. In addition, on the registered chromatogram of blank extracts did not appear specific peak for tigecycline (Figure 3).

### 3.3 Optimization of Liquid–Liquid Microextraction Procedures

The effect of microextraction parameters during tigecycline isolation from milk samples using deep eutectic solvents was studied on the basis of measured signal of analyte on registered chromatograms of the deep eutectic solvent extracts using the LC-MS/MS method. For this purpose, the mass ratio of DES components, volume of extractants, pH of extractive samples, shaking, and centrifugation time were optimized during liquid–liquid microextraction. The milk samples were deproteinizated using trichloroacetic acid solution (for DES decanoic acid:thymol and thymol:camphor) and acetonitrile for procedures using dodecanoic acid:menthol and dodecanoic acid:dodecanol as extraction media.

**Figure 4:**
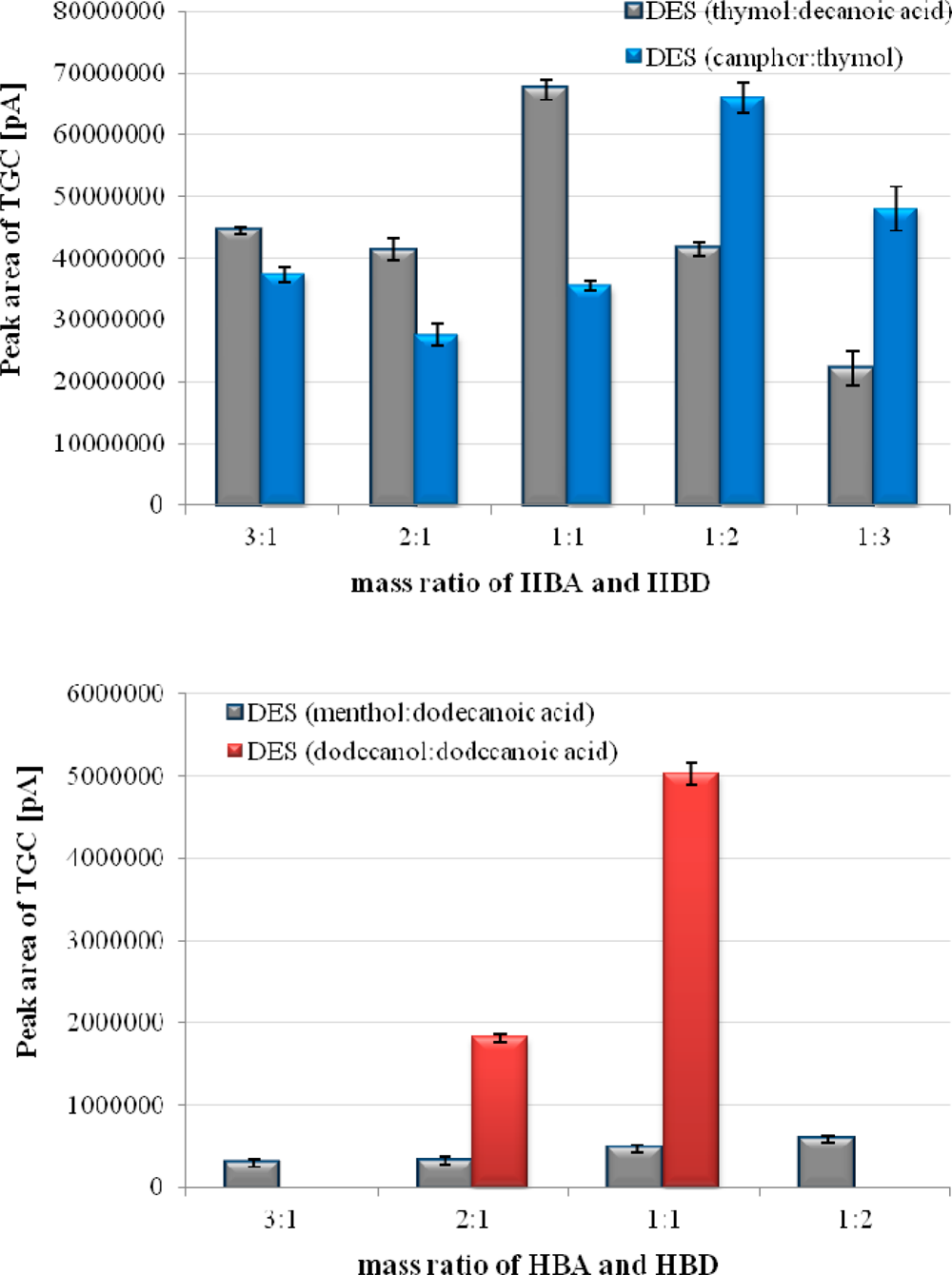
Effect of mass ratio of DES components on microextraction process of tigecycline (*n* = 3).

**Figure 5:**
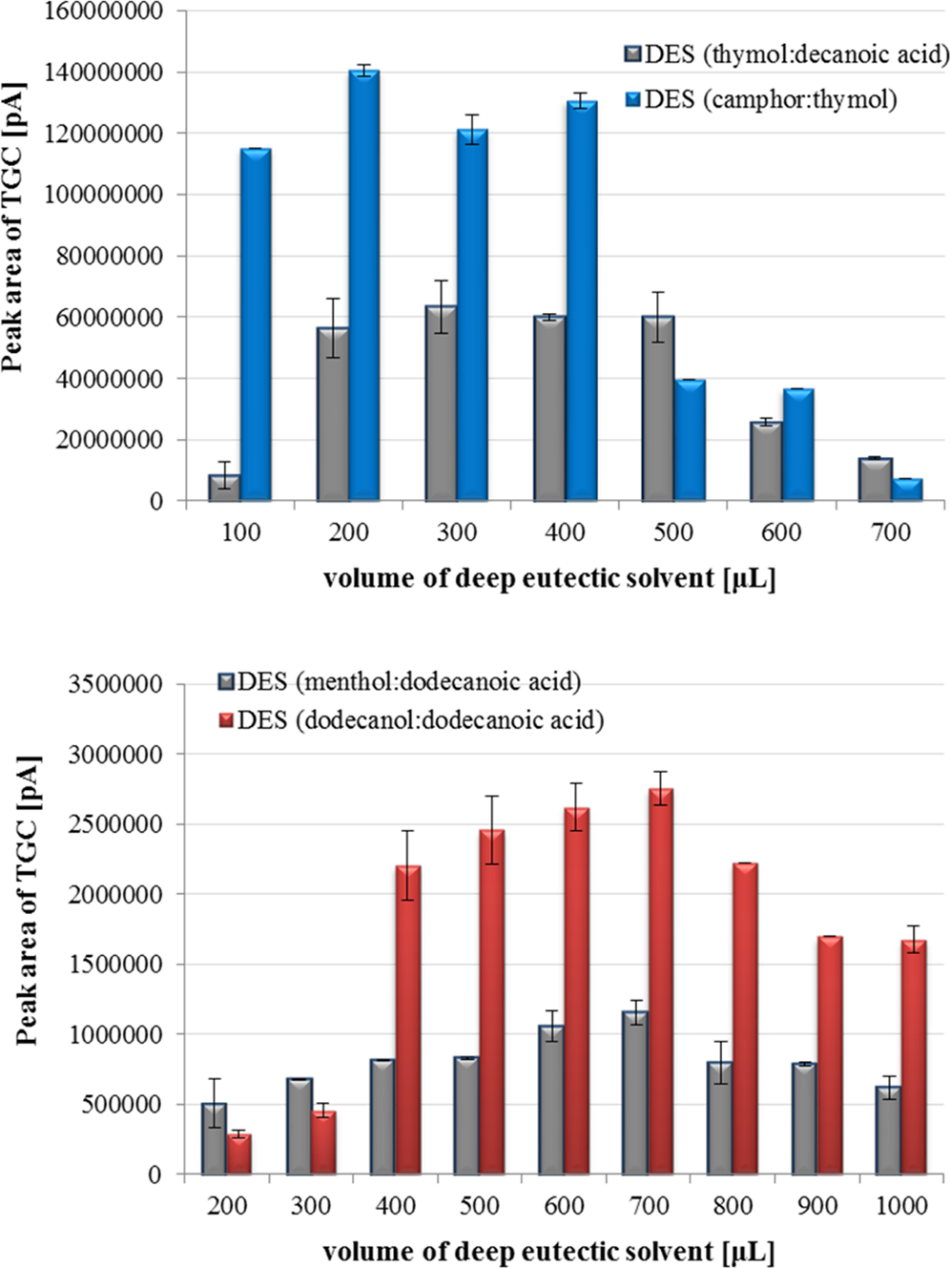
Effect of DES volume on liquid–liquid microextraction process of tigecycline with deep eutectic solvents (*n* = 3).

The mass ratio of deep eutectic solvent components plays an important role in the efficiency of the isolation method. Therefore, the microextraction procedure of tigecycline from the milk sample was performed using the different mass ratios of HBA and HBD in the applied deep eutectic solvents (3:1, 2:1, 1:1, 1:2, 1:3). The volume extractants during the microextraction process are very significant to achieve the effective isolation of the studied analyte. In the liquid–liquid microextraction process, the amounts of the used extractants are greatly reduced. Therefore, the influence of deep eutectic solvents volume for isolation of tigecycline was studied in the range 100–1000 μL. The peak area of analyte on the registered chromatograms as a function of mass ratio of DES components and DES amount was presented in Figure 4 and Figure 5.

It was observed that the isolation process of the studied compound was characterized by the low efficiency and incorrect phase separation after using DES in the mass ratio of components 3:1 or 1:3 (HBA:HBD). The results indicate that the highest efficiency of TGC microextraction was obtained for the following deep eutectic solvents: thymol and decanoic acid (1:1), camphor and thymol (1:2), menthol and dodecanoic acid (1:2), and dodecanoic acid and dodecanol (1:1). It was found that the measured signal of TGC was increasing with the increasing of the extractant volume up to 300 μL (thymol:decanoic acid), 200 μL (camphor:thymol), and 700 μL (menthol:dodecanoic acid, dodecanol:dodecanoic acid). The peak area of tigecycline in the obtained extracts was decreased above the mentioned deep eutectic solvents volume. Therefore, these amounts were chosen in subsequent experiments.

Tigecycline is the antibiotic from tetracycline class characterized by amphoteric properties.^32^ Therefore, the studied analyte exists as the ionic species in acidic and alkaline solution. The pH of the extractive sample containing TGC before the deproteinization process was equal to 6.4. This value favors the presence of a neutral form of the studied compound. The influence of the pH samples during the microextraction of tigecycline was investigated. The hydrogen ion concentration was changed using the addition of hydrochloric acid (0.1 mol L^–1^) and sodium hydroxide (0.1 mol L^–1^) solutions. The isolation process of TGC was performed in the pH range 3.0–10.0 of milk samples using the optimized deep eutectic solvents volume. It was found that the efficiency of DES-LLME procedures was unsatisfactory after the change of the pH of extractive samples. Moreover, the proper phase separation was difficult to achieve, and the characteristic peak of tigecycline on the registered LC-MS/MS chromatograms was deformed. After the addition of sodium hydroxide solution, the deproteinization process was also difficult and the microextraction of TGC was not possible at the pH sample range 8.0–10.0. Therefore, in further studies, the isolation process was performed without the change of pH extractive samples.

**Figure 6:**
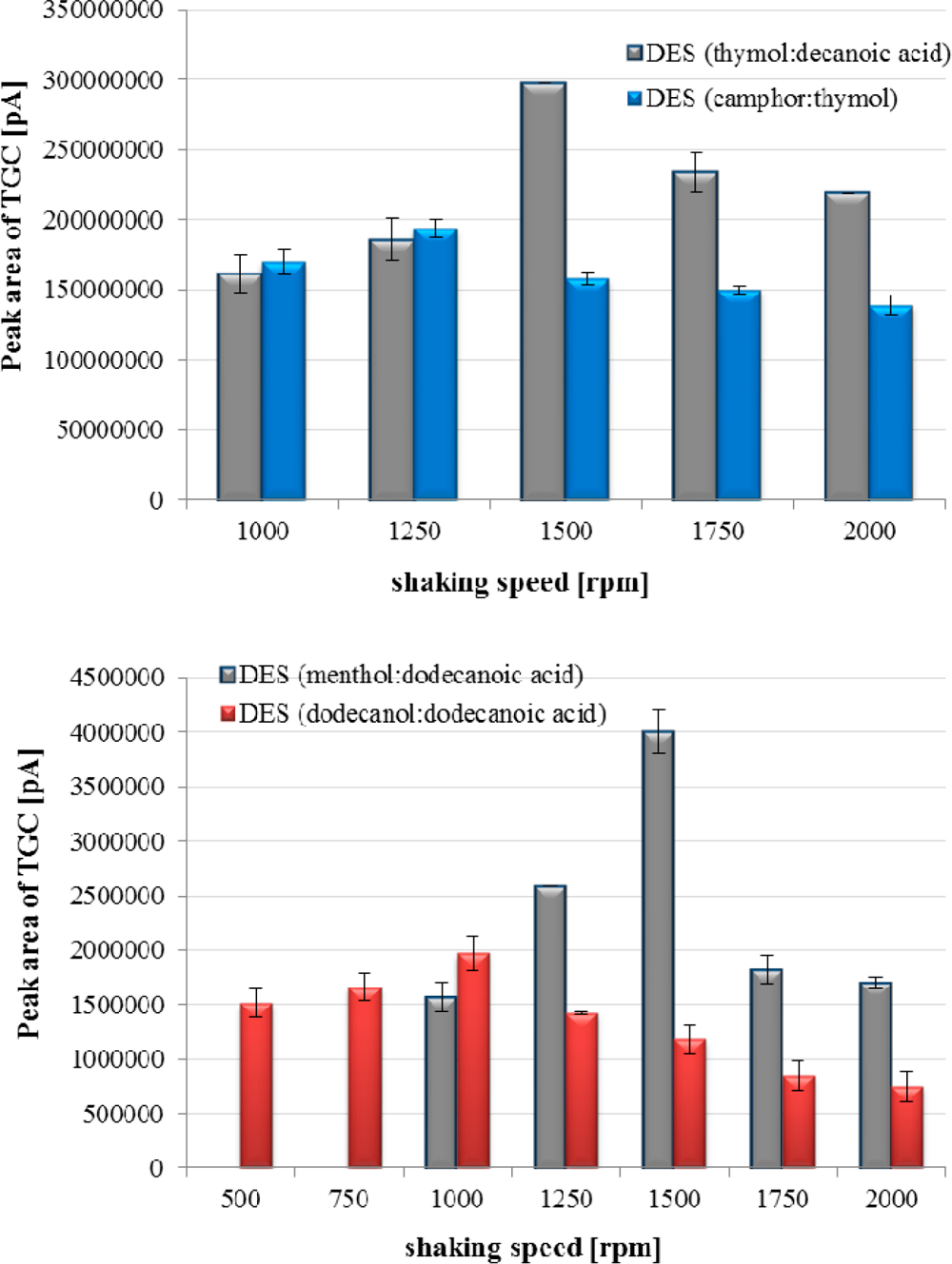
Influence of shaking speed on the measured signal of TGC using DES-LLME-LC-MS/MS procedure (*n* = 3).

**Figure 7:**
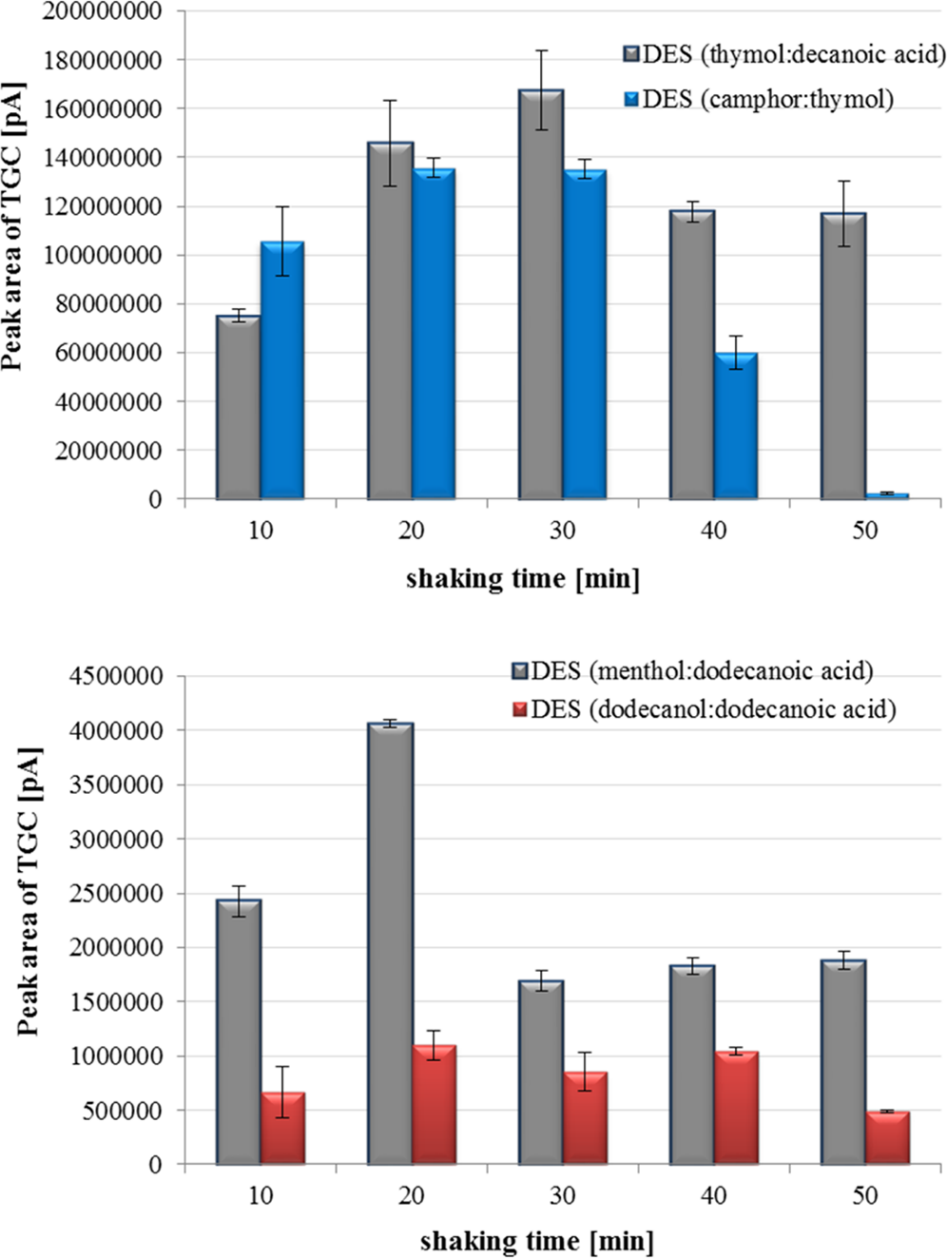
Influence of shaking time on the isolation processes of tigecycline from milk samples (*n* = 3).

The shaking process affects the efficiency of liquid–liquid microextraction by using hydrophobic deep eutectic solvents as extraction media. Therefore, the change in shaking speed values was studied during tigecycline isolation from milk samples. The miniaturized extraction of the analyte was performed using the variable shaking speed in the range 500–2000 rpm. The samples were shaken for 20 min. The obtained results of the measured signal of TGC as a function of the variable parameter values were presented in Figure 6. The effect of shaking time on samples during microextraction of TGC was also investigated. In this purpose, the isolation LLME process was performed using the selected shaking speed values and variable shaking time (10–50 min). The DES extracts were analyzed by chromatographic LC-MS/MS method and the obtained results were shown in Figure 7.

It was observed that the proper phase separation and satisfactory efficiency of the LLME process using shaking speeds of 500 and 750 rpm were only achieved for DES consisting of dodecanol and dodecanoic acid. It was found that the liquid–liquid microextraction of tigecycline from milk samples was the most effective after using a shaking speed of 1500 rpm for the following deep eutectic solvents, namely, thymol:decanoic acid and menthol:dodecanoic acid. This value was selected for the subsequent experiments while shaking speeds of 1250 and 1000 rpm were chosen during LLME isolation of tigecycline with DES thymol:camphor and dodecanol:dodecanoic acid, respectively. It was found that the peak area values of tigecycline increased with the increase of the shaking time up to 20 min (DES thymol:decanoic acid) and 30 min (DES thymol:camphor) and then decreased for the longer time. These values of the shaking time were selected for further studies. Whereas, during the use of deep eutectic solvents in liquid–liquid microextraction procedure consisting of dodecanoic acid the extractive samples should be shaken for 20 min in the subsequent experiments.

**Figure 8:**
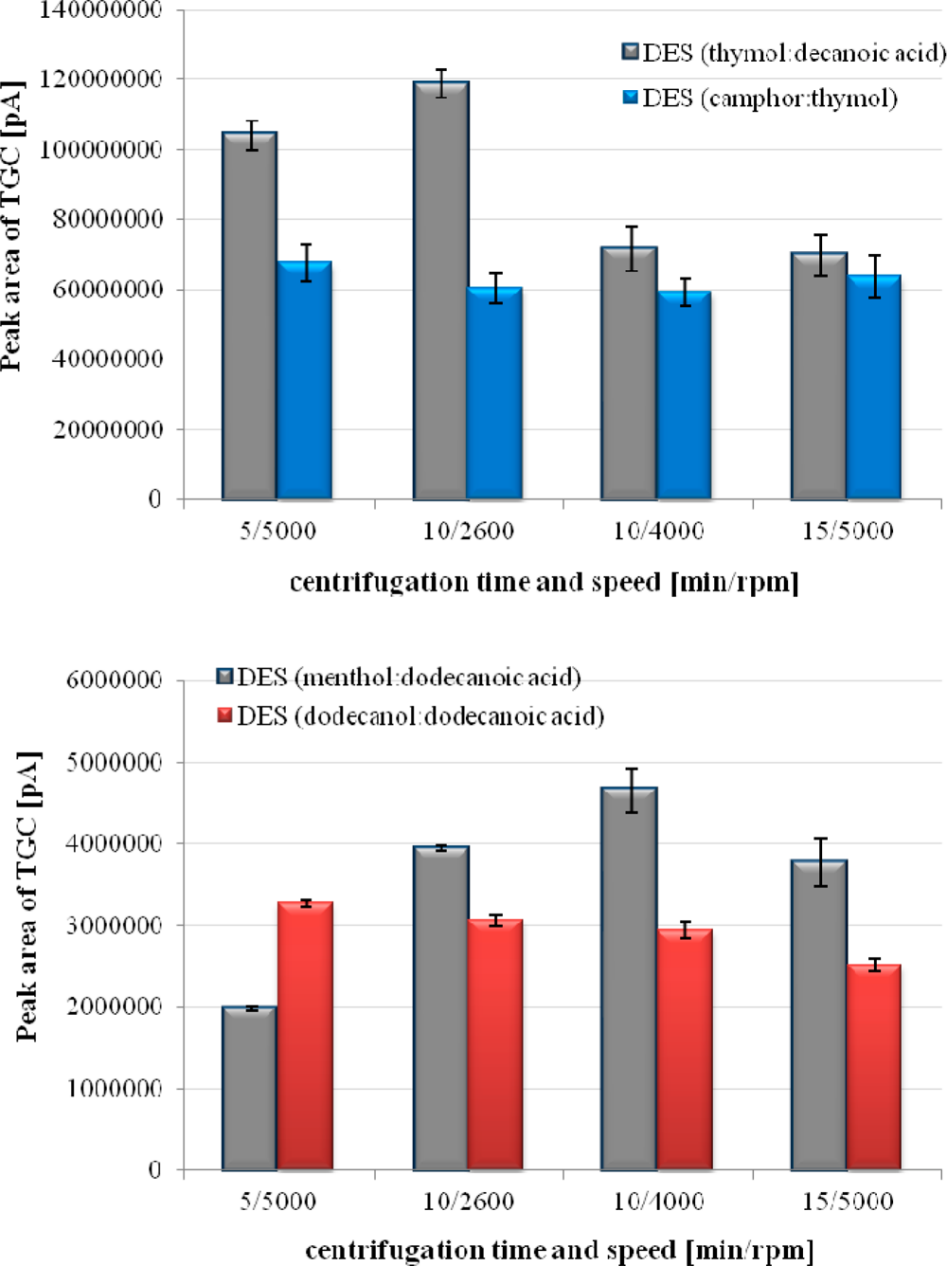
Influence of centrifugation process on the liquid–liquid microextraction of tigecycline using deep eutectic solvents (*n* = 3).

After the shaking process, the extractive samples were additionally centrifuged to achieve the proper phase separation between the deproteinizated milk sample and layer of deep eutectic solvent. The centrifugation process was performed at 5 min (5000 rpm) after LLME microextraction of TGC using extractants thymol:camphor and dodecanol:dodecanoic acid while the samples were centrifuged at 10 min (2600 rpm) for DES thymol:decanoic acid and at 10 min (4000 rpm) with using menthol:dodecanoic acid (Figure 8).

### 3.4 Validation of DES-LLME-LC-MS/MS Methods

The calibration curves of LC-MS/MS tigecycline determination after liquid–liquid microextraction using the optimal parameters and deep eutectic solvents as extractants were recorded. The deproteinization process of milk samples was performed using a trichloroacetic acid solution for microextraction with DES consisting of thymol. The analyte concentration range was 1 × 10^–10^ mol L^–1^ to 7 × 10^–5^ mol L^–1^ (thymol:decanoic acid) and 5 × 10^–9^ mol L^–1^ to 5 × 10^–5^ mol L^–1^ (thymol:camphor) (procedure 2.4.1), whereas the deproteinization process using acetonitrile solvent was performed before the LLME procedure with DES consisting of dodecanoic acid in the tigecycline concentration range 5 × 10^–8^ mol L^–1^ to 7 × 10^–5^ mol L^–1^ and 5 × 10^–9^ mol L^–1^ to 7 × 10^–5^ mol L^–1^ for dodecanoic acid:menthol and dodecanoic acid:dodecanol, respectively (procedure 2.4.2). The intraday precision of the developed methods was calculated by repeating microextraction procedures with deep eutectic solvents for TGC concentration of 3 × 10^–5^ mol L^–1^ (five samples in short period time), while the interday precision was estimated by the repeating tigecycline DES-LLME processes in the concentration range of the recorded calibration curves in a few successive days. The limit of detection (LOD) and limit of quantification (LOQ) values of the elaborated methods for tigecycline determination were estimated using the standard deviation of the lowest measured signal value and the slope of the calibration curve. The analytical parameters of DES-LLME-LC/MS/MS procedures were presented in Table 2. The obtained results indicated that the developed methods are characterized by a wide range of linearity and lower limit of detection and quantification values, especially for the microextraction using thymol and decanoic acid (LOD: 1.8 × 10^–11^ mol L^–1^ and LOQ: 5.5 × 10^–11^ mol L^–1^). The elaborated methods are distinguished by the satisfactory precision. The intraday parameter value was equal 1,4% for the tigecycline isolation using DES dodecanol:dodecanoic acid, and the interday precision for micorextraction of analyte with DES thymol:camphor was equal 5.4%. The average recovery of TGC was in the range 96.4–100.2%. The matrix effect was estimated during tigecycline determination using LC-MS/MS method after liquid–liquid microextraction with deep eutectic solvents. In this purpose, the matrix-matched and matrix-free calibration curves in the analyte concentration 5.0 × 10^–8^ mol L^–1^ to 5.0 × 10^–5^ mol L^–1^ were recorded. It was observed that the enhancement of TGC signal intensity based on the ratio of the slope of the calibration curves (matrix-matched to matrix-free) was less than 20%.

**Table 2:** Analytical Parameters of the Chromatographic (LC-MS/MS) Method for Tigecycline Determination Using the Microextraction Procedures with Deep Eutectic Solvents (*n* = 5)

	determination of tigecycline (DES-LLME-LC-MS/MS)			
analytical parameter	thymol:decanoic acid	camphor:thymol	menthol:dodecanoic acid	dodecanol:dodecanoic acid
retention time (min)	0.405	0.437	0.336	0.327
equation of calibration curve (*n* = 5)	*y* = 3.1 × 10^12^*x* + 23023	*y* = 1.2 × 10^12^*x* + 27736	*y* = 8.9 × 10^10^*x* + 7027	*y* = 1.4 × 10^11^*x* – 1708
slope ± standard deviation (SD)	3.1 × 10^12^ ± 3.5 × 10^11^	1.2 × 10^12^ ± 6.3 × 10^10^	8.9 × 10^10^ ± 6.8 × 10^9^	1.4 × 10^11^ ± 9.7 × 10^9^
coefficient of determination ± SD	*R*^2^ = 0.995 ± 0.007	*R*^2^ = 0.999 ± 0.001	*R*^2^ = 0.999 ± 0.002	*R*^2^ = 0.998 ± 0.003
linearity (mol L^–1^)	1 × 10^–10^ to 7 × 10^–5^	5 × 10^–9^ to 5 × 10^–5^	5 × 10^–8^ to 7 × 10^–5^	5 × 10^–9^ to 7 × 10^–5^
precision intraday (RSD) (*n* = 5) (%)	7.8	3.9	6.8	1.4
precision interday (RSD) (%)	11.7	5.4	7.6	7.1
LOD (mol L^–1^)	1.8 × 10^–11^	4.8 × 10^–10^	4.0 × 10^–9^	1.2 × 10^–9^
*(μg kg^–1^)	*0.01	*0.27	*2.28	*0.68
LOQ (mol L^–1^)	5.5 × 10^–11^	1.5 × 10^–9^	1.2 × 10^–8^	3.7 × 10^–9^
*(μg kg^–1^)	*0.03	*0.85	*6.84	*2.11
average value of recovery ± SD (*n* = 5) (%)	100.2 ± 5.7	96.4 ± 8.6	99.1 ± 3.1	98.8 ± 1.9

### 3.5 Comparison of the Elaborated Methods of Tigecycline Determination with the Described Procedures in the Literature

The elaborated procedures based on liquid–liquid microextraction with deep eutectic solvents and LC-MS/MS determination are more precise and characterized by the lower limit of detection and quantification values, wider range of linearity TGC concentration in comparison to methods described in the references^5,8,33−36^ (Table 3). The value of intraday parameter during determination of tigecycline using developed LLME-LC-MS/MS method was in the range 1.4–7.8%. This precision is more satisfactory than for the procedures presented in the references.^7,34^ The intraday parameter values during tigecycline determination in milk using chemiluminescence immunoassay and in rat bone samples with LC-MS/MS technique was within the ranges 6.1–8.5%^7^ and 3.6–10.7%.^34^ The use of deep eutectic solvents in microextraction process of the studied analyte before chromatographic analysis allows TGC detection in the concentration range 1.8 × 10^–11^ mol L^–1^ to 4.0 × 10^–9^ mol L^–1^ (0.01 ng mL^–1^ to 2.3 ng mL^–1^). These values are lower in comparison to the procedures described in the references.^5,37^ The limit of detection values during colorimetric determination of tigecycline in river water and fluoroimmunoassay for TGC analysis in egg sample were equal 4.46 × 10^–9^ mol L^–1^^5^ and 5.8 ng mL^–1^.^37^ The developed methods are also characterized by the lower LOQ values (0.03 ng g^–1^ to 6.84 ng g^–1^; 3.2 × 10^–5^ μg mL^–1^ to 7.0 × 10^–3^ μg mL^–1^) than the procedures presented in the literature.^38−40^ The limit of quantification values of tigecycline determination in human bone and plasma for methods in the mentioned references were equal 50 ng g^–1^ (LC-MS/MS),^38^ 0.05 μg mL^–1^ (UPLC-PDA),^39^ and 0.1 μg mL^–1^ (UPLC-MS/MS).^40^

**Table 3:** Comparison of the Elaborated DES-LLME-LC-MS/MS Method of Tigecycline Determination with the Procedures from the Literature

method	limit of detection (LOD)	limit of quantification (LOQ)
LC-MS/MS ^33^ (μg mL^–1^)	3 × 10^–3^	1.1 × 10^–2^
UHPLC-MS/MS ^35^ (mg L^–1^)	8.7 × 10^–2^ to 3.0 × 10^–1^	0.3–1.0
HPLC-MS/MS ^36^ (μg kg^–1^)	0.07	0.19

DES-LLME-LC-MS/MS (μg mL^**–1**^) (mg L^–1^)	1.0 × 10^–5^ to 2.3 × 10^–3^	3.2 × 10^-5^ to 7.0 × 10^–3^
(μg kg^–1^)	0.01–2.28	0.03–6.84

method	intraday precision	interday precision
UHPLC-MS/MS^35^ (%)	5–21	11–22
chemiluminescence immunoassy ^8^ (%)	8.6–9.8	7.6–12.7
DES-LLME-LC-MS/MS (%)	1.4–7.8	5.4–11.7

method	linearity
LC-MS/MS^34^ (ng g^–1^)	50 −10000
colorimetry^5^ (mol L^–1^)	2 × 10^–8^ to 6 × 10^–6^
DES-LLME-LC-MS/MS (ng g^-1^)	0.06–40000
(mol L^-1^)	1 × 10^–10^ to 7 × 10^–5^

## 4 Application of DES-LLME-LC-MS/MS Methods for the TGC Determination in the Different Milk Samples

The elaborated liquid–liquid microextraction procedures connected with chromatographic LC-MS/MS analysis was used for determination of tigecycline in bovine milk samples collected from the local markets (vanilla milk 1.5% w/w, lactose-free milk 2.0% w/w, milk 3.2% w/w, milk 0.5% w/w, and ecological milk 3.9% w/w). The deproteinization process of the spiked samples (TGC: 5 × 10^–7^ mol L^–1^ and 3 × 10^–5^ mol L^–1^) and their microextraction using deep eutectic solvents as extractants were performed according to procedures 2.3, 2.4.1, and 2.4.2. The obtained extracts containing tigecycline were analyzed by the LC-MS/MS method (procedure 2.5). The determined contents of the studied compound were presented in Table 4. It was observed that the phase separation process in the different sample matrices was difficult during use of extractants consisting of dodecanol and dodecanoic acid. Therefore, the analysis of milk 3.2% w/w, milk 0.5% w/w, and ecological milk was impossible using this kind of DES. The recovery of tigecycline in the analyzed samples was in the range 95.0–99.4% except ecological milk. The elaborated procedures DES-LLME-LC-MS/MS were additionally applied for analysis of real samples without a tigecycline standard. The obtained results indicated the absence of TGC in milk: vanilla, lactose-free, 3.2% w/w, and 0.5% w/w. The performed measurements give possibility of the studied antibiotic detection in the ecological milk sample at level 44.3 ± 0.2 (μg kg^–1^) including procedures with the following deep eutectic solvents thymol:decanoic acid, tymol:camphor and menthol:dodecanoic acid.

**Table 4:** Determination of Tigecycline in the Different Milk Samples Using DES-LLME-LC-MS/MS Procedures (*n* = 3)

procedure DES-LLME	added TGC concentration (mol L^–1^)	found TGC concentration (mol L^–1^) (*n* = 3)	average recovery ± RSD (%) (*n* = 3)	measured amount (μg kg^–1^] (*n* = 3)
Vanilla Milk (1.5% w/w)				
thymol:decanoic acid	5.0 × 10^–7^	4.82 × 10^–7^	96.5 ± 1.7	nd^a^
	3.0 × 10^–5^	2.84 × 10^–5^	94.7 ± 0.7	

thymol:camphor	5.0 × 10^–7^	4.91 × 10^–7^	98.2 ± 1.1	nd
	3.0 × 10^–5^	2.85 × 10^–5^	95.0 ± 1.9	

menthol:dodecanoic acid	5.0 × 10^–7^	4.97 × 10^–7^	99.3 ± 2.6	nd
	3.0 × 10^–5^	2.91 × 10^–5^	97.1 ± 2.9	

dodecanol:dodecanoic acid	5.0 × 10^–7^	4.87 × 10^–7^	97.4 ± 1.6	nd
	3.0 × 10^–5^	2.94 × 10^–5^	97.9 ± 1.9	
Lactose-Free Milk (2.0% w/w)				
thymol:decanoic acid	5.0 × 10^–7^	4.79 × 10^–7^	95.9 ± 1.7	nd
	3.0 × 10^–5^	2.98 × 10^–5^	99.3 ± 2.7	

thymol:camphor	5.0 × 10^–7^	4.91 × 10^–7^	98.2 ± 1.1	nd
	3.0 × 10^–5^	2.92 × 10^–5^	97.3 ± 2.0	

menthol:dodecanoic acid	5.0 × 10^–7^	4.84 × 10^–7^	96.9 ± 1.1	nd
	3.0 × 10^–5^	2.89 × 10^–5^	96.3 ± 6.1	

dodecanol:dodecanoic acid	5.0 × 10^–7^	4.90 × 10^–7^	98.1 ± 0.6	nd
	3.0 × 10^–5^	2.95 × 10^–5^	98.2 ± 1.2	
Milk (3.2% w/w)				
thymol:decanoic acid	5.0 × 10^–7^	4.84 × 10^–7^	96.8 ± 1.6	nd
	3.0 × 10^–5^	2.86 × 10^–5^	95.4 ± 3.0	

thymol:camphor	5.0 × 10^–7^	4.95 × 10^–7^	99.0 ± 0.9	nd
	3.0 × 10^–5^	2.90 × 10^–5^	96.8 ± 6.5	

menthol:dodecanoic acid	5.0 × 10^–7^	4.86 × 10^–7^	97.2 ± 0.5	nd
	3.0 × 10^–5^	2.98 × 10^–5^	99.4 ± 2.6	
Milk (0.5% w/w)				
thymol:decanoic acid	5.0 × 10^–7^	4.87 × 10^–7^	97.4 ± 0.8	nd
	3.0 × 10^–5^	2.96 × 10^–5^	98.7 ± 2.1	

thymol:camphor	5.0 × 10^–7^	4.90 × 10^–7^	98.1 ± 0.8	nd
	3.0 × 10^–5^	2.89 × 10^–5^	96.3 ± 0.7	

menthol:dodecanoic acid	5.0 × 10^–7^	4.77 × 10^–7^	95.3 ± 4.8	nd
	3.0 × 10^–5^	2.91 × 10^–5^	97.1 ± 5.3	
Ecological Milk (3.9% w/w)				
thymol:decanoic acid	5.0 × 10^–7^	5.78 × 10^–7^	115.7 ± 0.6	44.1
	3.0 × 10^–5^	3.01 × 10^–5^	100.3 ± 0.2	

thymol:camphor	5.0 × 10^–7^	5.79 × 10^–7^	115.8 ± 0.2	44.3
	3.0 × 10^–5^	3.01 × 10^–5^	100.3 ± 0.2	

menthol:dodecanoic acid	5.0 × 10^–7^	5.77 × 10^–7^	115.3 ± 0.3	44.6
	3.0 × 10^–5^	3.01 × 10^–5^	100.3 ± 0.2	

a nd (not detected).

## 5 Conclusions

The proposed methods of tigecycline determination as the new generation antibiotic from the tetracycline class were elaborated. The liquid–liquid microextraction as the miniaturized isolation process was applied connected with the modern extraction media: hydrophobic deep eutectic solvents (decanoic acid:thymol, thymol:camphor, dodecanoic acid:menthol, dodecanoic acid:dodecanol). The DES-LLME procedures enable the decreasing amount of organic solvents and effective isolation of tigecycline from milk samples. The connection of liquid–liquid microextration with LC-MS/MS method determination are characterized by a good precision of the measurements, wide range of concentration linearity, and low values of limits of detection and quantification, especially for the microextraction using DES consisting of thymol and decanoic acid (LOD: 1.8 × 10^–11^ mol L^–1^ and LOQ: 5.5 × 10^–11^ mol L^–1^). The intraday parameter value was equal 1.4% for the tigecycline isolation using DES dodecanol:dodecanoic acid, and the interday precision for micorextraction of analyte with DES thymol:camphor was equal to 5.4%. The average recovery of TGC after the isolation process was in the range 96.4–100.2%. The developed procedures gives possibility of tigecycline determination in the different milk samples using environmentally friendly methods at a low level concentration (0.01 μg kg^–1^ to 2.28 μg kg^–1^). The application of the elaborated methods for the analysis of the different milk samples allowed detection of the studied antibiotic in the ecological milk sample at a level of 44.3 ± 0.2 (μg kg^–1^). This content of tigecycline in the studied sample indicates the need for monitoring of antibiotics in the dairy food products.
